# Exome Sequencing and Linkage Analysis Identified Tenascin-C (*TNC*) as a Novel Causative Gene in Nonsyndromic Hearing Loss

**DOI:** 10.1371/journal.pone.0069549

**Published:** 2013-07-30

**Authors:** Yali Zhao, Feifan Zhao, Liang Zong, Peng Zhang, Liping Guan, Jianguo Zhang, Dayong Wang, Jing Wang, Wei Chai, Lan Lan, Qian Li, Bing Han, Ling Yang, Xin Jin, Weiyan Yang, Xiaoxiang Hu, Xiaoning Wang, Ning Li, Yingrui Li, Christine Petit, Jun Wang, Huanming Yang Jian Wang, Qiuju Wang

**Affiliations:** 1 Department of Otorhinolaryngology, Head and Neck Surgery, Institute of Otolaryngology, Chinese PLA General Hospital, Beijing, China; 2 BGI-Shenzhen, Shenzhen, China; 3 T-Life Research Center, Fudan University, Shanghai, China; 4 Key Laboratory of Mental Health, Institute of Psychology, Chinese Academy of Sciences, Beijing, China; 5 Department of Orthopaedic Surgery, Chinese PLA General Hospital, Beijing, China; 6 BGI-Tianjin, Tianjin, China; 7 School of Bioscience and Biotechnology, South China University of Technology, Guangzhou, China; 8 State Key Laboratory for AgroBiotechnology, College of Biological Sciences, China Agricultural University, Beijing, China; 9 Institute of Life Sciences, Chinese PLA General Hospital, Beijing, China; 10 Genetics and physiology of hearing, Neuroscience, College de France and Institute Pasteur, Paris, France; 11 Department of Biology, University of Copenhagen, Copenhagen, Denmark; Innsbruck Medical University, Austria

## Abstract

In this study, a five-generation Chinese family (family F013) with progressive autosomal dominant hearing loss was mapped to a critical region spanning 28.54 Mb on chromosome 9q31.3-q34.3 by linkage analysis, which was a novel DFNA locus, assigned as DFNA56. In this interval, there were 398 annotated genes. Then, whole exome sequencing was applied in three patients and one normal individual from this family. Six single nucleotide variants and two indels were found co-segregated with the phenotypes. Then using mass spectrum (Sequenom, Inc.) to rank the eight sites, we found only the *TNC* gene be co-segregated with hearing loss in 53 subjects of F013. And this missense mutation (c.5317G>A, p.V1773M ) of *TNC* located exactly in the critical linked interval. Further screening to the coding region of this gene in 587 subjects with nonsyndromic hearing loss (NSHL) found a second missense mutation, c.5368A>T (p. T1796S), co-segregating with phenotype in the other family. These two mutations located in the conserved region of *TNC* and were absent in the 387 normal hearing individuals of matched geographical ancestry. Functional effects of the two mutations were predicted using SIFT and both mutations were deleterious. All these results supported that *TNC* may be the causal gene for the hearing loss inherited in these families. *TNC* encodes tenascin-C, a member of the extracellular matrix (ECM), is present in the basilar membrane (BM), and the osseous spiral lamina of the cochlea. It plays an important role in cochlear development. The up-regulated expression of *TNC* gene in tissue repair and neural regeneration was seen in human and zebrafish, and in sensory receptor recovery in the vestibular organ after ototoxic injury in birds. Then the absence of normal tenascin-C was supposed to cause irreversible injuries in cochlea and caused hearing loss.

## Introduction

There are a total of 278 million people suffering from hearing loss in the world, while in China the number amounts to 2.78 million. More than 60% of cases can be attributed to genetic causes and inherited across generations. Approximately 70% of hereditary hearing loss is nonsyndromic and caused by monogenic mutations. About 22–25% of them is autosomal dominant nonsyndromic sensorineural hearing loss (ADNSHL) [1]. To date, more than 60 loci and 27 responsible genes for autosomal dominant hearing loss had been identified (hereditary Hearing Loss homepage, http://hereditaryhearingloss.org/). Most of these genes were identified through the traditional positional cloning and sequencing each gene in the critical interval one by one according to the gene function and expression pattern. The scope of this method however, is limited, especially in situations where there are a large number of genes in the mapped region. However, recent advances in exome sequencing (i.e., sequencing of all protein-coding regions of the genome) approach has shown the sensitivity and accuracy in the identification of the causal genes of several rare monogenic diseases such as Miller syndrome [2], especially in diseases inherited in recessive pattern. However, exome sequencing is an unbiased method to all genes. It adds the difficulties in identifying the heterozygous mutation responsible for autosomal dominant disease. Family-based linkage analysis could map the responsible gene in a special region, which is a more accurate compared to exome sequencing. Thus combinational strategy using linkage analysis and exome sequencing provides a powerful and affordable means to identify causative genes especially in autosomal dominant mode.

Here we report a five-generation Chinese family (family F013) with autosomal dominant hereditary hearing loss mapped on chromosome 9q31.3-34.3 (28.54 Mb) in 2004, where there are 398 predicted and reported genes. Six years later after gene mapped, we successfully identified a novel causative gene for this type of hearing loss in family F013, *TNC* (Tenascin-C, **NC**_000009.11, NM_002160.2, NP_002151.2), applying the combined strategy of linkage analysis and whole-exome sequencing. The causal role of this gene was supported by several lines: (I) the co-segregation of mutation with the phenotype; (II) its absence in 387 unaffected individuals of matched geographical ancestry; (III) the conservative nature of the mutation site; (IV) identification of another missense mutation of *TNC* in a two-generation Chinese hearing loss family; (V)*TNC* encoding protein, tenascin-C is a member of extracellular matrix (ECM) glycoprotein, and there have some reports about the pathological function of ECM proteins in hereditary hearing loss, such as Usherin [3] and cochlin [4]. (VI) Tenascin-C expresses under basilar membrane (BM) in cochlea, and is important for auditory development and self-recovery from injuries. BM proteins (laminin [5] and collagen 4 [6]) have been reported involved in hearing loss.

## Materials and Methods

### Ethics Statement

The study was approved by the Committee of Medical Ethics of Chinese People’s Liberation Army (PLA) General Hospital. We obtained a written informed consent from all the participants in this study. Written informed consent was obtained from the next of kin on the behalf of the minors/children participants involved in this study.

### Family Ascertainment and Clinical Diagnosis

**Figure 1:**
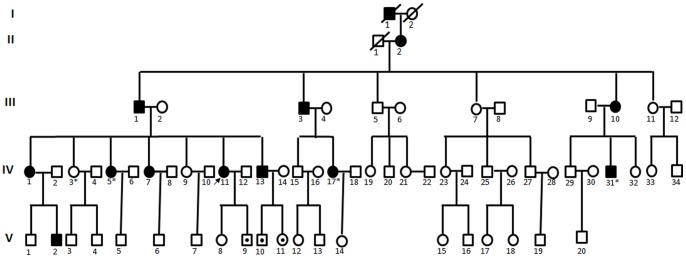
Pedigree of F013. Filled symbols for males (squares) and females (circles) represent affected individuals, and empty, unaffected ones. An arrow denotes the proband. Symbols with dot indicate the individual younger than the average age of onset, who are mutation carrier but does not present hearing loss (a mutation carrier).

A five-generation family (F013) with 70 members segregating ADNSHL was investigated from the Department of Otolaryngology, Head and Neck Surgery, the Chinese PLA (People’s Liberation Army) General Hospital, China (Figure 1). All participants underwent clinical and audiological evaluation, including physical examination, pure-tone audiometry, tympanometry, acoustic reflex, ABRs and DPOAE. The audiological data were evaluated based on the criteria established by European Working Group on Genetics of Hearing loss. High resolution computed tomography (HRCT) was also performed on some subjects to verify whether the family members had other complications other than hearing disorders. Environmental factors were also excluded as causes of hearing loss.

Peripheral blood samples were obtained and genomic DNA was extracted according to standard procedures, from a variety of affected and unaffected individuals. The first set of analysis was the five-generation Chinese hearing loss family, F013, a total of 22 subjects of which were included in the linkage study(II:2, III:1–5, III:7, III:9–11, IV:1–3, IV:5, IV:9, IV:11, IV:13, IV:17, IV:29, IV:31–32, V:1) and four individuals (IV:3, IV:5, IV:17, IV:31) were included in exome sequencing study (Figure 1). A cohort of 587 subjects with SNHL were chosen as the other affected set for further analysis. In these families the common gene associated to hearing loss, such as *GJB2*, *SLC26A4* and mitochondrial DNA A1555G mutations were all excluded. And 387 unaffected individuals of matched geographical ancestry were recruited for this study.

### Genotyping and Linkage Analysis

A genome-wide screening was performed with 394 microsatellite markers distributed with an average spacing of 10 cM intervals (ABI Prism Linkage Mapping Set 2 MD10, Applied Biosystems, Foster City, CA, USA). Additional markers for fine mapping of the linked chromosomal region were taken from the Marshfield chromosome 9 map (http://research.marshfieldclinic.org/genetics) and were also amplified using fluorescent-labeled primers (Table S1, Shenggong DNA Technologies, Shanghai China). Multiplex PCR was performed using standard procedures with PE9700 thermocyclers (Applied Biosystems), producing a final volume of 5 µl reaction mixture containing 30 ng of genome DNA, 1×PCR buffer, 0.2 mM of each dNTP, 3.0 mM MgCl_2_, 80 pmol of each forward and reverse primer, and 0.2 U of AmpliTaq Gold polymerase. PCR products were loaded onto a 6% denaturing polyacrylamide gel (7 M urea) and visualized on an ABI 3700 sequencer. Alleles were analyzed with ABI GeneMapper (version 3.0).

Two-point linkage between the disease locus and the markers was evaluated using the MILNK program of the LINKAGE software package (version 5.1). The disease was assumed to be an autosomal dominant disorder with a disease allele frequency of 0.0001 (an educated guess based on previous genetic epidemiological data). The allelic penetrance was set at 90% considering the genetic heterogeneity of hereditary hearing loss. For the microsatellite marker loci, equal allele frequencies were used. The genome-wide scan genotypic data of the 11 affected individuals were also analyzed using GeneHunter to obtain multipoint LOD scores. Haplotype analysis was constructed with the Cyrillic software version 2.1 (Cyrillic Software, Wallingford, UK).

### Exome Capture

Qualified genomic DNA samples (6 ug) of the F013 family members were sheared by sonication. Then the fragment of each sheared genomic DNA sample was hybridized to the SureSelect Biotiny lated RNA Library (BAITS) for enrichment.

### Exome Sequencing, Reads Mapping and SNP Detection

The enriched libraries were loaded on the HiSeq 2000 platform to be sequenced. Raw image files were processed by Illumina Pipeline v1.6 for base-calling with default parameters and the sequences of each individual were generated as 90 bp paired-end reads. And then the sequenced raw data was aligned to the the NCBI human reference genome (Build 36.3) using SOAPaligner [7]. After that, the duplicate reads were filtered out and the clean reads located in the target region were collected. Then the consensus genotype and quality were estimated by SOAPsnp (version1.03) using the clean reads. The low quality variations were filtered out by the following criteria: (i) quality score<20 (Q20); (ii) average copy number at the allele site> = 2; (iii) distance of two adjacent SNPs<5 bp; and (iv) sequencing depth<4 or >500.

### Detection of Insertions and Deletions

Insertions and deletions (indels) in the exome regions were identified through the sequencing reads. We aligned the reads to the reference genome by Burrows-Wheeler Aligner (BWA0.5.8 ) [8], and passed the alignment result to the Genome Analysis Toolkit (GATK1.0.4705) to identify the breakpoints. Finally, we annotated the genotypes of insertions and deletions [9].

### Sequencing Analysis of Candidate Gene

As the candidate gene of F013, *TNC* gene was selected for mutation screening in members available of F013, 587 subjects with SNHL and the 387 unaffected individuals. *TNC* gene contains 28 exons. Thirty-two primer pairs were designed using online Primer 3.0 software and synthesized by Invitrogen by life technology (Table S2, Beijing, China) to amplify each exon and exon-intron boundaries. PCR was performed with PE9700 thermocyclers (Applied Biosystems). The reaction mixture contained 100 ng DNA, 1.5 units of DNA Taq polymerase (TaKaRa, Dalian, China), 200 µM dNTPs, 3 pmol of each forward and reverse primer, 2.5 µL of 10× buffer (with 2.5 mM MgCl_2_) and the final reaction volume was filled to 25 µL with ddH_2_O. After PCR amplification, 5 µl PCR products were separated on 1% agarose gel and purified using Millipore filter plates. Sequence analysis was performed on an automated sequencer (ABI 3730, Applied Biosystems) for both affected and normal individuals of the family. Nucleotide alterations including mutations and polymorphisms were identified by sequence alignment with the NCBI *Reference Sequence* (Build 36.3) using the DNAStar software 5.0 version (DNASTAR, Madison, WI, USA).

## Results

### Family Recruitment and Clinical Features

**Figure 2:**
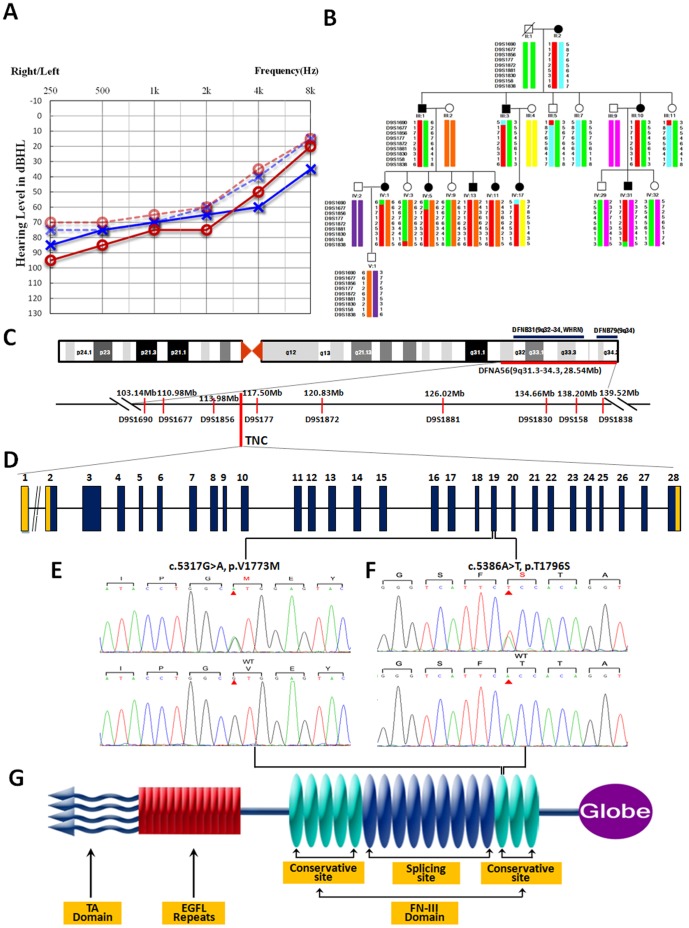
Gene mapping of F013 with autosomal dominant progressive hearing loss and audiological evaluation of affected family members. (A) Audiograms of the proband (IV:11). Symbols “o” and “x” denote air conduction pure-tone thresholds at different frequencies in the right and left ear. dB, decibels; Hz, Hertz. The proband is most affected in low frequencies with ascending audiological curve. The age at the time of audiological examination with dotted line was 18 years old. Audiological examination with solid line was performed ten years later. The hearing loss progresses gradually with age. (B) Haplotype analysis of F013. Haplotypes for the markers on chromosome 9q31.3-34.3 are shown by bars, with the haplotype associated with hearing loss in red which defines the critical DFNA56 region between flanking markers D9S1677 and D9S1838. (C) Schematic physical and genetic maps of DFNA56 locus on the 9q31.3-34.3 chromosomal region. The previously reported loci, DFNB31 and DFNB79 which are overlapped with DFNA56 are also shown. The *TNC* gene is indicated. Mb, million base pairs. (D) Structure of *TNC* gene. *TNC* gene has 28 exons. Mutations of c.5317G>A (p.V1773M) and c.5368A>T (p.T1796S) identified in *TNC* gene are both located in exon 19, which corresponds to the 13th FN-III domain in the translated protein. (E) & (F) Sequencing chromatograms of *TNC* showing the two heterozygous transitions, c.5317G>A and c.5368A>T in affected individuals (upper panel) compared with those of normal controls (lower panel). The two mutated nucleotides are marked by red arrows. The predicted amino acid changes and surrounding ones are indicated above the sequences. (G) Tenascin-C is composed of a TA domain, a linear array of epidermal growth factor-like (EGFL) repeats, a series of fibronectin type III (FN-III) domains, and a globular domain at the terminal.

*A total of* 53 *family members,* composed of *11 clinical affected* and 42 unaffected individuals (37 presumptive unaffected family members *and 5 clinically unknown subjects younger than the average onset age), were recruited in this study (* *Figure 1* *).* Affected members in F013 showed a postlingual, symmetrical, and bilateral nonsyndromic sensorineural hearing loss (Figure 2A). The hearing loss was initially presented as mild in low frequencies with subsequent gradual progression to severe level involving all frequencies with time (Figure 2A). Age of onset varied from 8 to 30 years old. No vestibular symptoms or signs *were* reported. High resolution CT scan showed normal *middle* and *inner* ears structure, including normal vestibular aqueduct and internal auditory canal. Comprehensive examination of the family medical history did not identify any other clinical syndromic feature.

### Linkage Analysis

Twenty-two of the 53 subjects in F013 were enrolled in the linkage study (Figure 2B). The genome-wide linkage analysis of F013 located the deafness gene on the long arm of chromosome 9. Significant linkage was found with markers D9S164 and D9S1826 (positive two-point LOD scores of 3.44 and 3.31 at  = 0, respectively, Table 1) within the chromosomal region 9q31.3-34.3 (Figure 2C). Fine mapping of the region using additional microsatellite markers (Table S1) confirmed the linkage. The maximum two-point LOD score of 4.57 ( = 0) was obtained with marker D9S177 in chromosomal region 9q33 (Table 1). This candidate region showed partial overlap with the critical intervals identified in DFNB31 (9q32-34) and DFNB79 (9q34.3) loci (Figure. 2C), both of which inherited in autosomal recessive pattern. Thus, the critical interval mapped on chromosome 9q31.3-34.3 in this study is a novel DFNA locus, assigned as DFNA56 by Human Genome Organization (HUGO) nomenclature committee (http://www.gene.ucl.ac.uk/nomenclature/).

**Table 1:** Two-point LOD scores between 9q microsatellite markers for F013.

Markers	Genetic	Physical	LOD SCORE AT *θ* =						Z_max_	*θ*_max_
	Distance(cM)	Distance(bp)	0	0.1	0.2	0.3	0.4	0.5		
D9S1690	106.63	103143785	−13.39	−1.03	−0.05	0.27	0.25	0	0.29	0.344
D9S1677	117.37	110981204	−2.1	2.86	2.38	1.68	0.83	0	2.93	0.061
D9S1856	120.04	113986910	3.11	2.63	2.1	1.49	0.81	0	3.11	0
D9S289	120.77	115455388	4.31	3.6	2.81	1.92	0.94	0	4.31	0
D9S1824	122.23	115931926	0.97	0.81	0.64	0.45	0.24	0	0.97	0
D9S1776	123.33	116999255	2.55	2.07	1.54	0.94	0.34	0	2.55	0
D9S177	123.87	117499409	4.57	3.82	2.99	2.05	1.01	0	4.57	0
D9S154	125.63	118380667	2.84	2.36	1.83	1.22	0.56	0	2.84	0
D9S1872	129.74	120829322	4.27	3.57	2.79	1.91	0.93	0	4.27	0
D9S1116	130.52	122032251	2.58	2.14	1.65	1.1	0.49	0	2.58	0
D9S1682	132.09	124033006	2.38	2.08	1.69	1.22	0.67	0	2.38	0
D9S1881	135.85	126019300	3.44	2.87	2.23	1.51	0.72	0	3.44	0
D9S1840	136.47	126327340	2.58	2.1	1.55	0.95	0.33	0	2.58	0
D9S290	140.86	130567273	1.11	0.94	0.75	0.53	0.29	0	1.11	0
D9S1795	142.51	131346313	1.63	1.33	0.99	0.62	0.23	0	1.63	0
D9S1861	144.13	132310567	2.88	2.39	1.85	1.23	0.56	0	2.88	0
D9S1830	145.21	134655582	4.31	3.6	2.81	1.92	0.94	0	4.31	0
D9S164	147.91	135195754	3.44	2.87	2.23	1.51	0.71	0	3.44	0
D9S1826	159.61	137551425	3.31	2.92	2.35	1.63	0.8	0	3.31	0
D9S158	161.71	138202283	0.02	0.02	0.01	0.01	0	0	0.02	0
D9S1838	163.84	139519106	−3.19	1.49	1.36	0.99	0.47	0	1.5	0.115

### Exome Sequencing Identified a Candidate Gene

Three affected individuals (IV:5, IV:17, IV:31) and one normal hearing (IV:3) member of F013 were included in whole exome sequencing study. We obtained an average of 4.7 billion bases of sequence per individual as paired-end, 90 bp reads, and about 65% of the total bases mapped to the exomes with a mean coverage of 50-fold (Table S3, Figure S1 and Figure S2). At this depth of coverage, 98% of the targeted region was sufficiently covered to pass our thresholds for variant calling. By exome sequencing, we detected 240 coding insertions or deletions (Indels). To identify SNPs, we used SOAPsnp software and bioinformatics pipeline. A total of 7,580 non-synonymous/splice acceptor and donor site/insertions or deletions (NS/SS/Indel) variants were detected in at least one of the affected individuals (Table S4 and Table S5). After comparison with SNP databases, including dbSNP132, the 1000 Genome Project (10/2010 release), HapMap data (HapMap 8/2010 release), and YH (Asian), we identified 6 rare SNPs and 2 indels in transcribed sequences that co-segregated with the phenotype in these 4 subjects (Table 2). Among them, a missense mutation c.5317G>A (p.V1773M) in exon 19 of the *TNC* gene, is just located in the critical linked region of 9q31.3-34.3 (Table 3). We screened the whole exon19, where the variant located in *TNC*, in all 53 members of F013 using Sanger sequencing. Results found that all 11 patients were heterozygous for this mutation and none of the clinically unaffected family members carried this variant, which is co-segregated with the hearing loss phenotype (Figure 2E). Furthermore, six candidate SNPs and 2 indels in transcribed sequences found in exome sequencing were also genotyped using the Sequenom MassARRAY platform (Sequenom, Inc.) in all family members obtained. Except the mutation c.5317G>A in *TNC*, none of the other seven variants were co-segregated with the hearing loss.

**Table 2:** Identification of the causal gene by whole-exome sequencing and linkage analysis.

Filter process	IV:5(Whole/locus)	IV:17(Whole/locus)	IV:31(Whole/locus)	IV:5+IV:17(Whole/locus)	IV:5+IV:17+IV:31(Whole/locus)
NS/SS/Indel	7515/160	7483/170	7401/165	5071/130	4081/120
Not in dbSNP 132_1000 Genomes_HapMap_YH	540/10	524/9	486/9	130/4	51/3
Not in dbSNP 132_1kgenomes_Hapmap_YH_control	303/3	398/6	387/7	48/2	8/1

The number of functional variants (non-synonymous/splice acceptor and donor site/insertions or deletions) is listed under various filters. Variants were filtered by presence in dbSNP, 1000 Genomes, HapMap 8 or YH (Not in dbSNP 132_1000 Genomes_HapMap_YH) and control exomes (Not in dbSNP_1kgenomes_Hapmap_YH_control). (Whole/locus): indicate NS/SS/Indel variants be observed in whole exome or in locus region by linkage analysis.

**Table 3:** Candidate variants shared by three affected individuals of F013.

Chromosome	Position	Reference	Change	Gene	Codons	Substitution
Chr6	151714872	A	T	*AKAP12*	AAA-AtA	K1218I
Chr6	152671466	A	–	*SYNE1*	Splice site	deletion
Chr7	151601810	G	T	*MLL3*	CCT-tCT	P309S
Chr9	116843116	C	A	*TNC*	GTG-aTG	V1773M
Chr10	51282920	T	G	*TIMM23*	AGA-gGA	R112G
Chr16	28511211	C	T	*SULT1A2*	GAG-tAG	E217X
Chr17	77866494	A	C	*CD7*	TAC-cAC	Y239H
Chr19	45786926	GAGGACAGTCCTGTCCAACAGGGAGG		*SHKBP1*	Splice site	deletion

Multiple sequence alignment in other organisms (including human, macaque, chimpanzee, elephant, pig, platypus and zebrafish) revealed the mutation was present in a highly conserved position (Figure S3). We assessed the functional impact of p.V1773M on the protein using SIFT (version 4.0.3) and Align-GVGD software, and this mutation was predicted to be functionally deleterious. We used PolyPhen-2 software (DeLano Scientific LLC) to study 3D structure of the protein, and found that mutation (p.V1773M) changes the shape of the protein in this region, which may alter its ability to bind to other molecules. This mutation was located in an exon that was present in all RNA isoforms. There is no total length of tenascin precursor in Protein Data Bank (PDB), and we took the FN-III domain structure in rat that is similar to *TNC* sequence as the template (PDB: 1TDQ, PubMed: 15296743, Sup. file 1) After comparison, the mutation located in the rim of conjunction of two conservative domains in FN-III domain, which was the most functional part of tenascin-C for ligand binding (Figure S4).

### Mutation Spectrum of the *TNC* Gene

To confirm the results, we sequenced the whole 28 exons of *TNC* gene in 587 subjects with SNHL that have unknown genes. We found a second mutation in a three-generation Chinese hearing loss family (Family 6957 with 5 patients; Figure S5) in exon19 of *TNC*, which was a missense variant c.5368A>T transition (p. T1796S) (Figure 2F) and completely co-segregated with the hearing loss phenotype in all 5 patients. Hearing loss in this family was late onset and the high frequency was initially decreased (Figure S6). It’s interesting that three patients (II:1, II:8, II:12) also carried the mitochondrial mutation of 1555A>G. Additionally, we found 15 non-synonymous variants in heterozygous pattern (Table S6 and Table S7). These variants were rare and carried by one or two individuals only, nine of them were novel variants which have never be reported in public database (Table S7). SIFT predicted that four variants from the nine was damaging. Details of six patients with the four *TNC* variants were described in the supplementary materials (Table S8). To assess the frequency of the mutation with p.V1773M and p.T1796S in population, we sequenced 387 geographically matched controls and neither mutation was found in the samples at present.

## Discussion

The F013 has been mapped to 9q33 by linkage analysis six years ago by our team, but the causative gene was not identified because of too many candidate genes involved in this mapped locus. This caveat is common and unconquerable in the study of hereditary hearing loss because of the intrinsic defect of traditional method. Now, about 80 loci in 155 for nonsyndromic sensorineural hearing loss (NSSHL) including 30 dominant NSSHL loci remain to be identified. Strong power of exome sequencing technology in identifying causative gene has been exemplified for monogenic disease [2], and gradually for common diseases [10]. Previous study showed that this new technique is especially fit for diseases in recessive inheritance mode, because in domiant inherited mode the heterozygosity will be causative and be clinically effective and too many functional SNPs would be identified. However, exome sequencing is unbiased to detect all functional variants in coding region. After filteration by public data and familial controls, damaging mutations emerged. Then, by the help of exact location of linkage analysis, redundant mutations will be excluded and we could detect the causal mutation underlie F013. Using this strategy, combination the mapped interval by classical linkage analysis with results of exome sequencing, only one causative mutation were confirmed. The combination of linkage analysis and exome sequencing provided strong evidence that *TNC* may be responsible for hearing loss in this family. The causative evidence was elaborated in introduction and concrete procedures could be seen in methods and results.

*TNC* gene encodes an extracellular matrix protein and has a highly conserved sequence in different species, for example, the sequence originated from the mouse has about 74% human similarity and the zebrafish is about 72%. The gene product tenascin-C has a structure like hexabrachion, composing of a TA domain, a linear array of epidermal growth factor-like (EGFL) repeats, a series of fibronectin type III (FN-III) domains, and a globular domain at the terminal (Figure 2G) [11]. The most important functional part of the protein is the FN-III domains. We used Protein Data Bank (PDB) archive to predict the protein structure change of Tenascin-C with mutations. We found that both mutations located in the rim the conjunction of two conservative domains in FN-III domain, which was the most functional part of tenascin-C for ligand binding. Mutation p.V1773M changes the shape of the protein in this region and tightens the distance of two adjacent FN-III domains, which may alter its ability to bind to other molecules (Figure S4). Although p.V1773M was recorded in dbSNP as a known variant, it is a rare SNP with very low frequency as shown in the ssSNP report (http://www.ncbi.nlm.nih.gov/projects/SNP/snp_ss.cgi?ss=ss342281564). The actual ratio was below 0.0005, and the number of subjects found by NCBI was 2 (2/4552 = 0.0004393). The similar artifact was also seen in the study from Walsh. They found a pathogenic mutation in dbSNP, but not in 768 controls. The reason was thought to be the use of uncurated variant database [12].

Tenascin-C, a glycoprotein, a member of the extracellular matrix (ECM), was expressed in the basilar membrane and the osseous spiral lamina in the human cochlear, which is one of the primary components in mammalian basilar membrane as collagen 4, laminin, nidogen, and dystroglycan. Because the basilar membrane in the inner ear regulates fluid and ion transport between the endolymph and perilymph [13], mutated components of the basilar membrane could lead to disturbance of ionic homeostasis and result in hearing loss, as shown by laminin [5] in nonsyndromic deafness and an altered expression of collagen IV chains in Alport’s syndrome [6]. And other proteins in ECM have also been shown to be involved in hereditary hearing loss, for example, mutated Usherin caused Usher syndrome IIA and mutated collagen XI caused nonsyndromic deafness [14].

Tenascin-C managed the recovery of sensory receptors in vestibular organ in birds after ototoxic injury [15]. The ability of spontaneous recovery after spinal lesion in adult zebrafish could also be explained by the promotion of tenascin-C [16]. And some studies in human have also conducted the conclusion that tenascin-C play an important role in the tissue repair and restoration in skeletal system [17], and in tissues remodeling after myocardial injury [18]. Considering the remarkable sequence conservation in different species, it is speculated the similar impulse in repairment in human cochlea. Under mutated conditions, the altered protein couldn’t bind to other extracellular matrix proteins, ligands and cell receptors, and the repair mechanism after reversible injuries would not work. The vulnerabilities of cochlea caused the accumulation of detriment and the hearing loss progressed.

In summary, using a combination of linkage analysis and exome-sequencing enabled us to use a single pedigree to identify a novel hearing loss gene. These findings suggest the usefulness and decreased cost that whole exome-sequencing can provide for gene identification in hereditary NSHL and other dominant Mendelian diseases. Additionally the identification of *TNC* here adds new evidence for the importance of BM proteins in hereditary hearing loss.

## Supporting Information

Figure S1 **The distribution of per-base sequencing depth in target regions for each sample.** Y-axis indicated the percentage of total target region under a given sequencing depth.
(TIF)

Figure S2 **Cumulative depth distribution in target regions for each sample.** X-axis denotes sequencing depth, and y-axis indicated the fraction of bases that achieves at or above a given sequencing depth. From the figure above, we can see about 75.50% of target region bases obtains at least 20× fold coverage, that is to say, about 75.50% of target region was covered by more than 20 reads. And about 89.10% of target region achieved at least 10×.
(TIF)

Figure S3 **Multiple amino acid sequence alignment of TNC using ClustalW software.** The conservation analysis shows that p.V1773M (red arrow) and p.T1796S (red arrow) heterozygous missense mutation in TNC is at a highly conserved position by comparison to the corresponding sequence of human, macaque, chimpanzee, elephant, pig, platypus and zebrafish.
(TIF)

Figure S4 **Comparison of normal and mutated protein configuration of tenascin precursor.** The image in cyan is the configuration prediction of normal protein. Sites of p.1773V and p.1796T are in purple. The image in green is the configuration prediction of mutated protein. Mutated sites of p.1773M and p.1796S are in yellow. And local zoom of normal and changed (p.V1773M) protein configuration emphasized by yellow circles.
(TIF)

Figure S5 **Pedigree of 6957.** Filled symbols for males (squares) and females (circles) represent affected individuals, and empty, unaffected ones; An arrow denotes the proband.
(TIF)

Figure S6 **Audiograms of the patients in family 6957.** Symbols “o” and “x” denote air conduction pure-tone thresholds at different frequencies in the right and left ear, symbol “□”denote marked air conduction pure-tone threshold with white noise. dB, decibels; Hz, Hertz. The age at the time of audiological examination was recorded.
(TIF)

Table S1 **Details of the 14 Markers used for fine mapping.**
(DOCX)

Table S2 **Primer sequences for each exon in TNC.**
(DOCX)

Table S3 **Summary of Effective Data for exome sequencing.** *The region near target refers to flanking region within 200 bp of target regions. **Total effective reads is the same meaning as the unique mapped reads which was stated in the pipeline above. Here the effective reads consist of two parts: i) the reads have only one best hit in the alignment. These reads comes from the unique region of genome ii) the reads have multiple best hits on the genome (the number of hits between 1 and 20), and they were randomly aligned onto the target regions. These reads mainly come from low complex genomic region, such as repetitive sequences, and account for about 4% of total effective reads. ***Target regions used here refer to genomic regions that the Exome array actually covered. The aggregate length of target is about 37.8 Mb.
(DOCX)

Table S4 **Summary of SNPs in Exome Sequencing for each Sample.** *Consensus genotype with quality score of at least 20. **Intronic SNPs within 4 bp of exon/intron boundary. ***5′ UTR refers to 200 bp upstream of initiation codon, 3′UTR is defined as 200 bp downstream of termination codon.
(DOCX)

Table S5 **Summary of Indels in Exome Sequencing for each Sample.**
(DOCX)

Table S6 **Nonsynonymous Variants of TNC in NSHL subjects.**
(DOCX)

Table S7 **Nine Novel Nonsynonymous Variants of TNC detected in NSHL subjects.**
(DOCX)

Table S8 **Details of patients carried the deleterious novel variants.**
(DOCX)
