# Supplementary figures and images for: Exome Sequencing and Linkage Analysis Identified Tenascin-C (TNC) as a Novel Causative Gene in Nonsyndromic Hearing Loss
Source: PLoS One. 2013 Jul 30;8(7):e69549. doi: 10.1371/journal.pone.0069549 (PMC3728356; doi:10.1371/journal.pone.0069549)

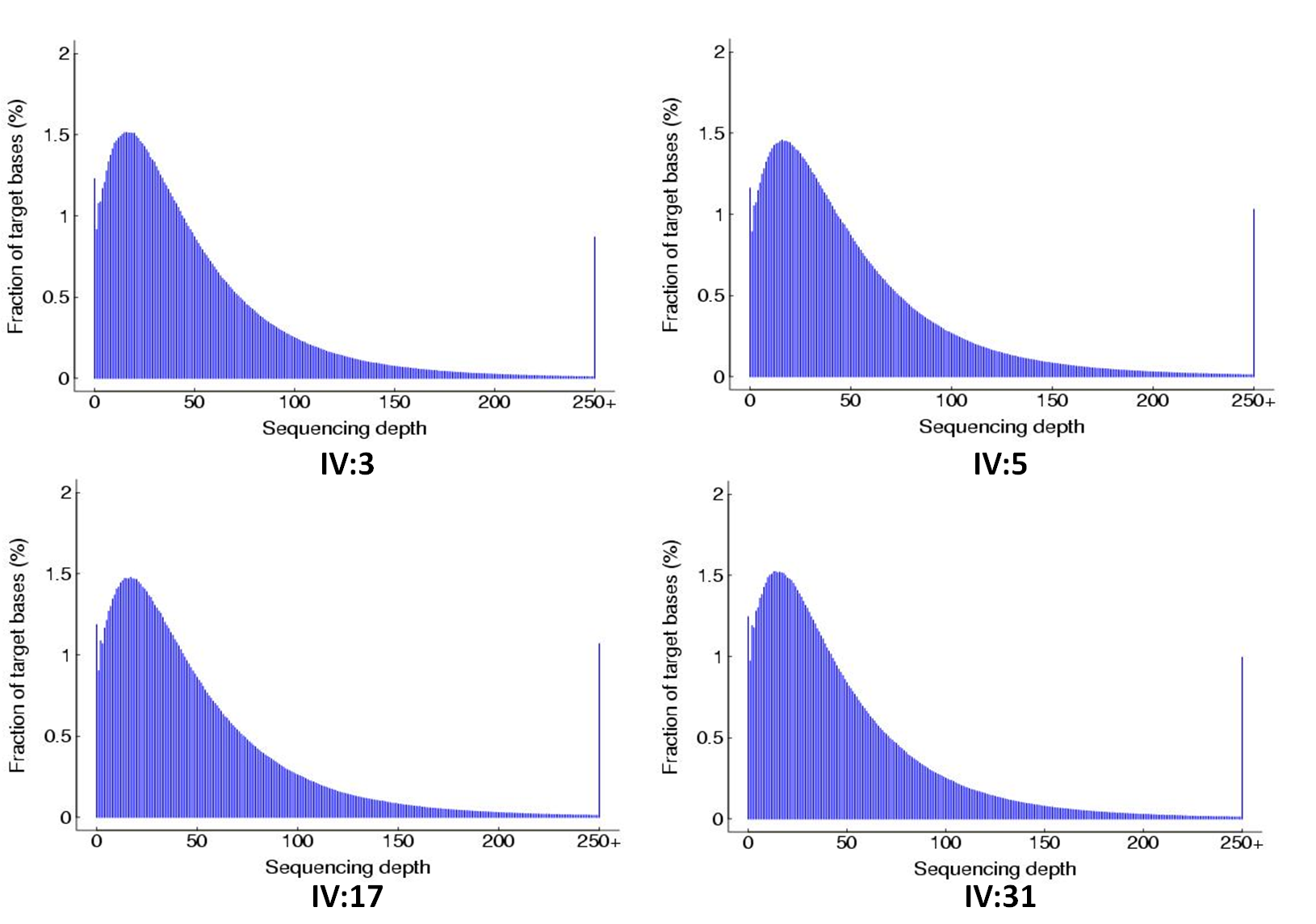

Supplement: Figure S1 — The distribution of per-base sequencing depth in target regions for each sample. Y-axis indicated the percentage of total target region under a given sequencing depth. (TIF) [file pone.0069549.s001.tif]

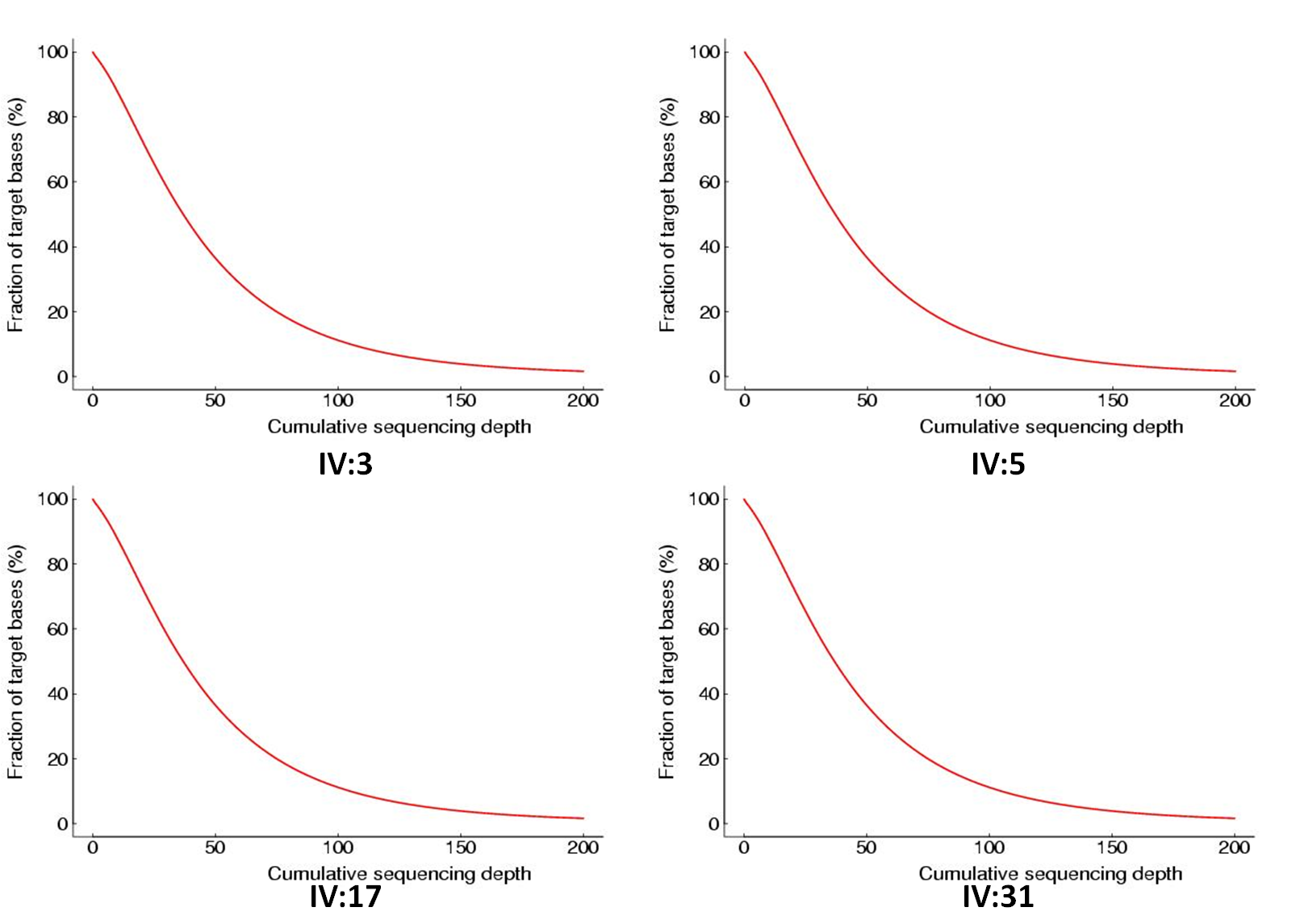

Supplement: Figure S2 — Cumulative depth distribution in target regions for each sample. X-axis denotes sequencing depth, and y-axis indicated the fraction of bases that achieves at or above a given sequencing depth. From the figure above, we can see about 75.50% of target region bases obtains at least 20× fold coverage, that is to say, about 75.50% of target region was covered by more than 20 reads. And about 89.10% of target region achieved at least 10×. (TIF) [file pone.0069549.s002.tif]

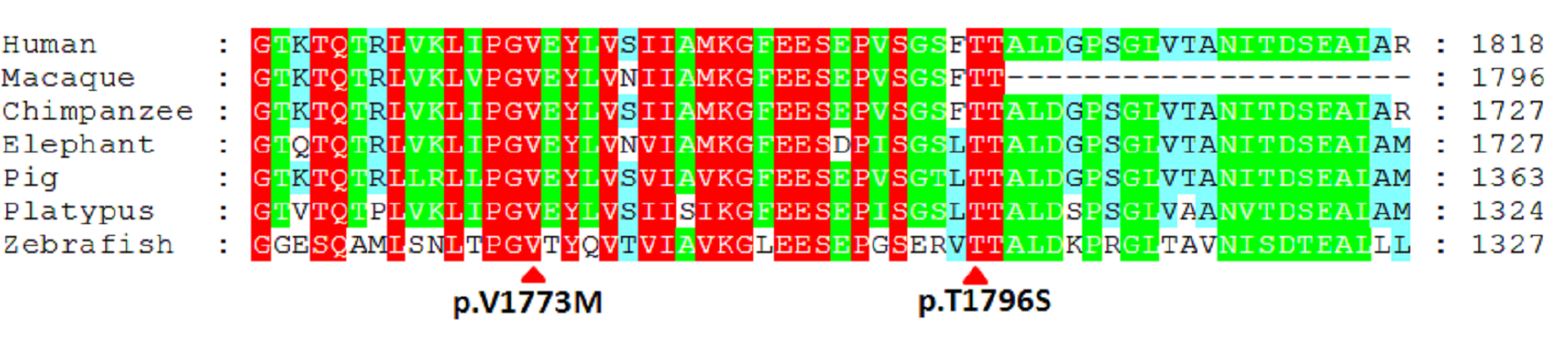

Supplement: Figure S3 — Multiple amino acid sequence alignment of TNC using ClustalW software. The conservation analysis shows that p.V1773M (red arrow) and p.T1796S (red arrow) heterozygous missense mutation in TNC is at a highly conserved position by comparison to the corresponding sequence of human, macaque, chimpanzee, elephant, pig, platypus and zebrafish. (TIF) [file pone.0069549.s003.tif]

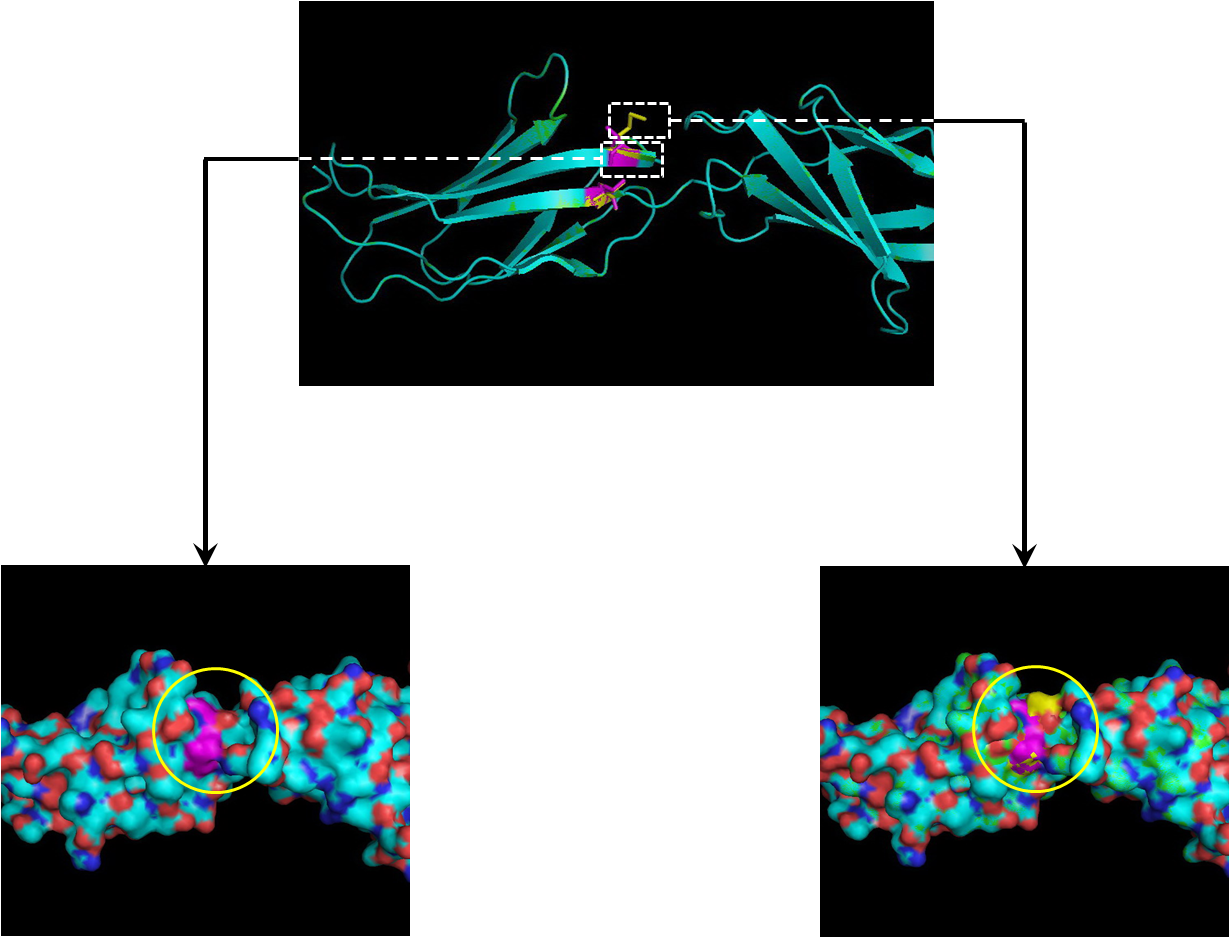

Supplement: Figure S4 — Comparison of normal and mutated protein configuration of tenascin precursor. The image in cyan is the configuration prediction of normal protein. Sites of p.1773V and p.1796T are in purple. The image in green is the configuration prediction of mutated protein. Mutated sites of p.1773M and p.1796S are in yellow. And local zoom of normal and changed (p.V1773M) protein configuration emphasized by yellow circles. (TIF) [file pone.0069549.s004.tif]

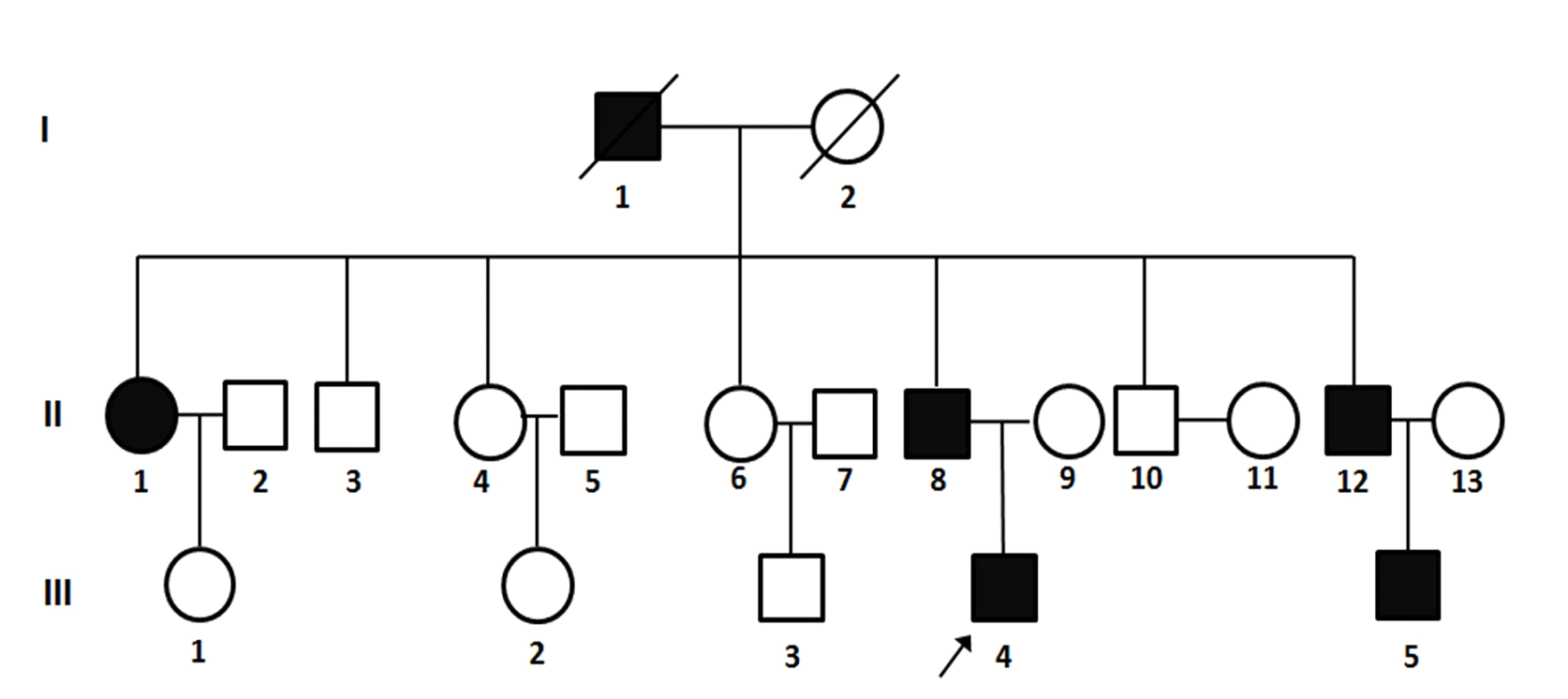

Supplement: Figure S5 — Pedigree of 6957. Filled symbols for males (squares) and females (circles) represent affected individuals, and empty, unaffected ones; An arrow denotes the proband. (TIF) [file pone.0069549.s005.tif]

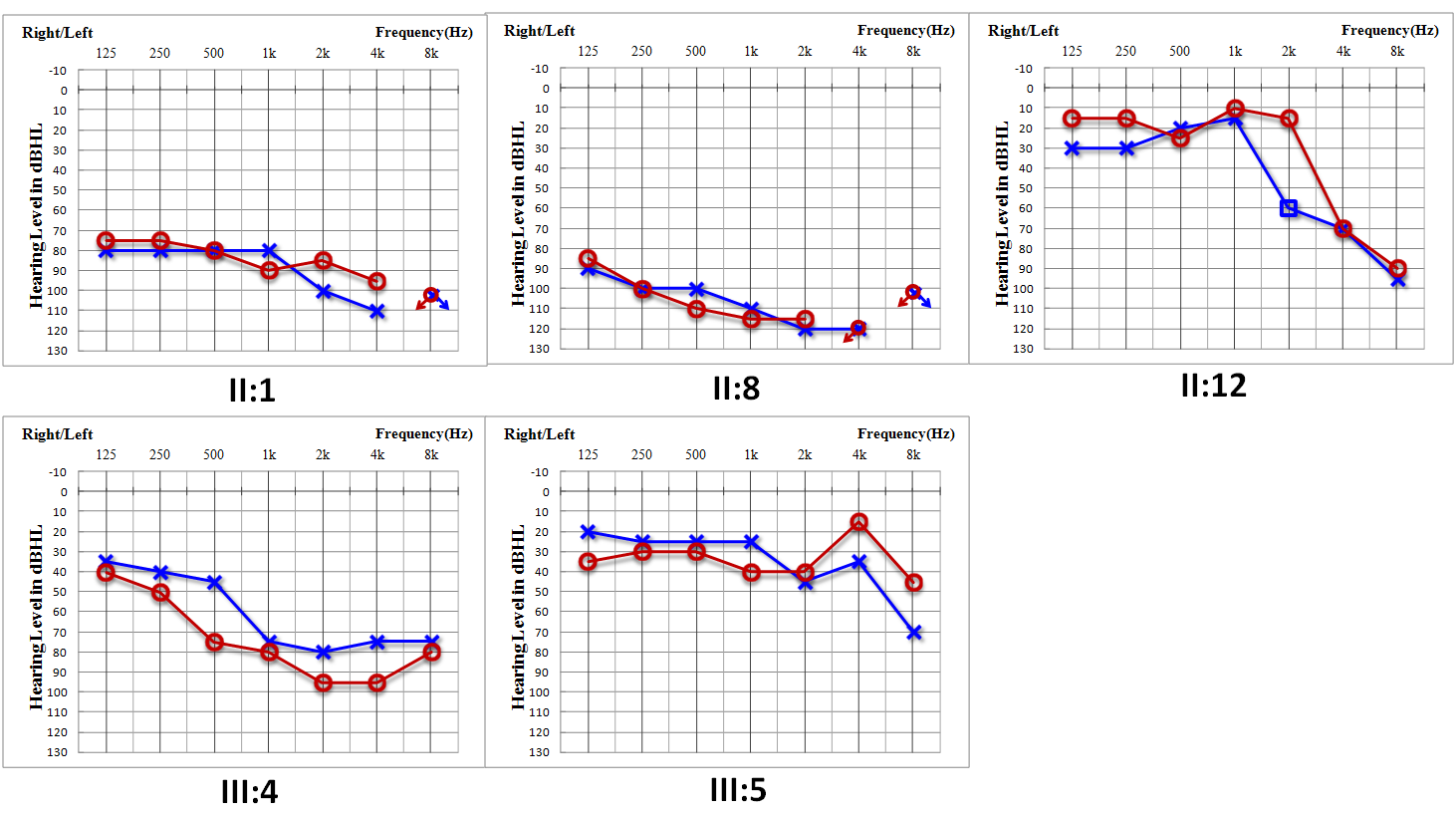

Supplement: Figure S6 — Audiograms of the patients in family 6957. Symbols “o” and “x” denote air conduction pure-tone thresholds at different frequencies in the right and left ear, symbol “□”denote marked air conduction pure-tone threshold with white noise. dB, decibels; Hz, Hertz. The age at the time of audiological examination was recorded. (TIF) [file pone.0069549.s006.tif]
