# Supplementary material for: Exome Sequencing and Linkage Analysis Identified Tenascin-C (TNC) as a Novel Causative Gene in Nonsyndromic Hearing Loss
Source: PLoS One. 2013 Jul 30;8(7):e69549. doi: 10.1371/journal.pone.0069549 (PMC3728356; doi:10.1371/journal.pone.0069549)
Supplement: Table S1 — Details of the 14 Markers used for fine mapping. (DOCX) [file pone.0069549.s007.docx]

**Table S1 Details of the 14 Markers used for fine mapping**

| **Locus** | **Het** | **ASR** | **Genotyper** | **Primers sequence(5’ to 3’, Forward)** | **Primers sequence(5’ to 3’, Reverse)** |
| --- | --- | --- | --- | --- | --- |
| D9S1856 | 0.86 | 231–252 | 248-244 | GGATTTGGGAGCAAACTTCAGAC | TTGTGGAATAAAAACACCAACAGC |
| D9S289 | 0.75 | 75–87 | 79-79 | TGCATTAGAAAACCTTCCA | CCCAGCCTATTTGCAT |
| D9S1824 | 0.79 | 118–128 | 118-128 | GTGTTCTCCAGAGAGACAGAGC | TCCTAATTGTGTGAGCCAATG |
| D9S177 | 0.86 | 211–237 | 219-233 | CAGGGGTCAGAATCTTAAAG | CCCTTCATACAAAAACTACCAC |
| D9S154 | 0.85 | 139–171 | 165-165 | ATAAAGTGGAGTCGGACAGG | GAGTCAGGATTTGAACCCAG |
| D9S1872 | 0.89 | 111–135 | 115-117 | TAGAAACTGGGGCAGG | TGGGAATCTGTGGAGG |
| D9S1116 | 0.74 | 405–421 | 413-413 | TGCATGATTTTCTGAGATGG | GACACTTTCACATGTCTTACCG |
| D9S1881 | 0.83 | 220–236 | 224-230 | GGCCCAACACCAATGTCAC | CAATGAGATCATCCATGCAGAGC |
| D9S1840 | 0.57 | 110–122 | 116-120 | ACCAATCAGAAACCTTGCC | TTAAGAACAGAAGCGCATAGGAG |
| D9S1795 | 0.72 | 130–146 | 142-146 | CCTGGGCGACAAAGTG | GCCAAAGCGTGATTTTTTA |
| D9S1831 | 0.81 | 239–267 | 255-263 | CAGTGAATCGAGGTCGC | CCAGTGTTAAAGTCAGCCG |
| D9S1861 | 0.73 | 182–194 | 182-190 | GATTGCTTGAGCCCAGG | AAGGAGCTTCTTCACAACCC |
| D9S1830 | 0.74 | 107–117 | 113-117 | CAGAGTGGTGGGACTCAA | AGCTGCAGACTGCCTTC |
| D9S1838 | 0.82 | 159–175 | 159-163 | ACCCAGCTACTGAGGAGGCTT | GCTTCTGCACTTTGTAGAACCAAAT |
