# Supplementary material for: Exome Sequencing and Linkage Analysis Identified Tenascin-C (TNC) as a Novel Causative Gene in Nonsyndromic Hearing Loss
Source: PLoS One. 2013 Jul 30;8(7):e69549. doi: 10.1371/journal.pone.0069549 (PMC3728356; doi:10.1371/journal.pone.0069549)
Supplement: Table S2 — Primer sequences for each exon in TNC. (DOCX) [file pone.0069549.s008.docx]

**Table S2 Primer sequences for each exon in TNC**

| **EXON** | **Product length (bp)** | **GC%** | **Primers sequence(5’ to 3’, Forward)** | **Primers sequence(5’ to 3’, Reverse)** |
| --- | --- | --- | --- | --- |
| Exon1 | 569 | 49.7 | CCAGGAGTGAGTGCGTCT | CGAAAAGTTCCTTCCAAGTCT |
| Exon2 | 860 | 50.1 | GAGAGCTTCAAATACAGGGAT | GTCTCCACACTTATTTGAACAT |
| Exon3-Part 1 | 633 | 55.3 | CTGGTATAACAGGATGTGAGTCT | CTCATCACACACGCACTCAT |
| Exon3-Part 2 | 523 | 58.1 | ACCAGGGCAAGTGCGTAAAT | GCTCCAGTGAAACCATCAT |
| Exon3-Part 3 | 619 | 59.1 | CTGCTACTGCGAAGAAGGCT | GTCATTTGGACAGGAGAGTT |
| Exon3-Part 4 | 650 | 54.5 | AGGCTTCAAGGGCTATGACT | AAAAGTCATTATGGGTGTGT |
| Exon4 | 518 | 53.7 | GAACAGCCACCACAGGTCT | GCGAGGGAGGGTTATACACT |
| Exon5 | 482 | 41.3 | ATTCCAAAGCCTGCGTTCAT | CTTGTCCTCCACCAAAAACT |
| Exon6 | 472 | 43 | ACGAATGGGAGAAACAAACT | GCAGCAAAAGTTAAATGATGT |
| Exon7 | 520 | 50.2 | TCAGAAAGGCAAAGTAAAGT | CCTTTCATTTTTCGTACTGT |
| Exon8 | 502 | 48 | GCCAAATCTGTAGCACTTGT | GCAATCCAATAAGCAGTAT |
| Exon9 | 378 | 45 | CCCTCAGAAATAAGTCCAAT | TTTTCCTCCAGGCTTCTACT |
| Exon10 | 487 | 55.2 | AAAGAGAGACCCAGAGATGT | ACACCCTGGCTGAGGAGAT |
| Exon11 | 538 | 51.3 | CATTGTTGTCCTGCTGGTGT | CATTCCAAAGCTAGTCGTGTCT |
| Exon12 | 486 | 46.7 | CCTCTCTTTGCTCCATTTCT | AGAGACAAAGGGGAGGAAGT |
| Exon13 | 461 | 52.9 | GCTCAACACCTGCTGTAAAT | GCTGGCTCCTATCATGTCT |
| Exon14 | 458 | 52.6 | TCCCCTTTCTCTCTTTCCT | GTTATGGCGAGATGCTGACT |
| Exon15 | 478 | 43.3 | GGTAACATTGCTTCCTTCCAT | CACCCTGCAAAGAAGACTGT |
| Exon16 | 478 | 48.3 | TGAATGATGGACTTGGGTAT | GGGAATGTGAACAAAGATGT |
| Exon17 | 520 | 45.6 | CTTTCCCCTCTCCTGACTCT | TGAGAAGAAGACCCCTGAGT |
| Exon18 | 296 | 48.6 | GGCTTGGCTCAGACACAAAT | CCATCCAAACCCACAACACT |
| Exon19 | 321 | 45.5 | AAGTGGTGTTTGATGAAGAT | CAAGTGACCTCTGAAAAATAGT |
| **EXON** | **Product length (bp)** | **GC%** | **Primers sequence(5’ to 3’, Forward)** | **Primers sequence(5’ to 3’, Reverse)** |
| Exon20 | 339 | 52.5 | GGCTCCAGGCTTAGGTCTCT | GAAGAGCGAATGGGAAGAGT |
| Exon21 | 338 | 52.4 | CGCAGGGTCACTGAAAGT | CTAGCGGTTCCTGTTTAAGAT |
| Exon22 | 378 | 45.8 | GGCTCAACCACCTTATTTAT | GAAAAACAGCATCAGGTAAT |
| Exon23 | 378 | 44.7 | GAATGGATGGGTAAATGAACT | TCCTCAGGCTTCCAAGTAGT |
| Exon24 | 404 | 50.5 | GATTGCCCATTTCTATGAGTT | AGAGTGCATCAGGAGGAGAT |
| Exon25 | 361 | 47.1 | ACCCAAAGTTACATAGCAAAT | CTGCCACCAGAAGTAGTTC |
| Exon26 | 403 | 52.4 | CCTCCCAAAGTGCTGAGAT | AATGAGGAAGGAATGAAAGTT |
| Exon27 | 414 | 51.2 | TTCAATGCTGACTCTGGGAT | GTTCCTGCCTCCTAGTCTCT |
| Exon28-Part 1 | 587 | 46.3 | ACCCAGACCAACAGAGAGAT | CCAGAGCCACCTAAGAGAAGT |
| Exon28-Part 2 | 637 | 36.7 | CTGGGCATTTGGTGAGAGT | CCACTTGACCACTATCCCTACT |
