# Supplementary material for: Exome Sequencing and Linkage Analysis Identified Tenascin-C (TNC) as a Novel Causative Gene in Nonsyndromic Hearing Loss
Source: PLoS One. 2013 Jul 30;8(7):e69549. doi: 10.1371/journal.pone.0069549 (PMC3728356; doi:10.1371/journal.pone.0069549)
Supplement: Table S4 — Summary of SNPs in Exome Sequencing for each Sample. *Consensus genotype with quality score of at least 20. **Intronic SNPs within 4 bp of exon/intron boundary. ***5′ UTR refers to 200 bp upstream of initiation codon, 3′UTR is defined as 200 bp downstream of termination codon. (DOCX) [file pone.0069549.s010.docx]

**Table S4 Summary of SNPs in Exome Sequencing for each Sample**

| **SNPs found in Exon Capture** | **IV:3** | **IV:5** | **IV:17** | **IV:31** |
| --- | --- | --- | --- | --- |
| Number of genomic positions for calling SNPs: | 106194180 | 106194180 | 106194180 | 106412297 |
| Number of high-confidence genotypes*: | 71304339 | 72282474 | 69327484 | 69050194 |
| Number of high-confidence genotypes in TR: | 36445186 | 36426874 | 36418414 | 36442556 |
| Number of known SNP site in TR: | 154403 | 154403 | 154403 | 154403 |
| Coverage of population SNPs in TR: | 145128 (93.99%) | 145213 (94.05%) | 144399 (93.52%) | 144957 (93.88%) |
| Total number of SNPs: | 38959 | 39238 | 37618 | 36777 |
| Synonymous-coding: | 8529 | 8470 | 8462 | 8434 |
| Missense: | 7064 | 7040 | 6988 | 6960 |
| Nonsense: | 45 | 46 | 55 | 44 |
| Readthrough: | 8 | 8 | 9 | 7 |
| Splice site**: | 321 | 336 | 338 | 321 |
| Intron: | 20473 | 20805 | 19316 | 18638 |
| 5' UTRs***: | 1093 | 1125 | 1096 | 1068 |
| 3' UTRs: | 1275 | 1241 | 1219 | 1158 |
| Intergenic: | 151 | 167 | 135 | 147 |

* Consensus genotype with quality score of at least 20.

** Intronic SNPs within 4bp of exon/intron boundary.

*** 5' UTR refers to 200bp upstream of initiation codon, 3'UTR is defined as 200bp downstream of termination codon.
