# Supplementary material for: Exome Sequencing and Linkage Analysis Identified Tenascin-C (TNC) as a Novel Causative Gene in Nonsyndromic Hearing Loss
Source: PLoS One. 2013 Jul 30;8(7):e69549. doi: 10.1371/journal.pone.0069549 (PMC3728356; doi:10.1371/journal.pone.0069549)
Supplement: Table S5 — Summary of Indels in Exome Sequencing for each Sample. (DOCX) [file pone.0069549.s011.docx]

**Table S5 Summary of Indels in Exome Sequencing for each Sample**

| **Indels found in Exon Capture** | **IV:3** | **IV:5** | **IV:17** | **IV:31** |
| --- | --- | --- | --- | --- |
| Total mumber of indels | 2416 | 1735 | 2264 | 2163 |
| Ins-coding | 76 | 67 | 65 | 63 |
| Del-coding | 119 | 107 | 117 | 123 |
| Splice site | 62 | 43 | 58 | 62 |
| Intron | 1932 | 1343 | 1827 | 1719 |
| 5' UTRs | 104 | 74 | 73 | 80 |
| 3' UTRs | 110 | 89 | 108 | 104 |
| Intergenic | 13 | 12 | 16 | 12 |
| Total insertion | 1104 | 775 | 1034 | 981 |
| Total deletion | 1312 | 960 | 1230 | 1182 |
| Heterozygous indels | 1261 | 902 | 1211 | 1150 |
| Homozygous indels | 1155 | 833 | 1053 | 1013 |
