# Supplementary material for: Exome Sequencing and Linkage Analysis Identified Tenascin-C (TNC) as a Novel Causative Gene in Nonsyndromic Hearing Loss
Source: PLoS One. 2013 Jul 30;8(7):e69549. doi: 10.1371/journal.pone.0069549 (PMC3728356; doi:10.1371/journal.pone.0069549)
Supplement: Table S6 — Nonsynonymous Variants of TNC in NSHL subjects. (DOCX) [file pone.0069549.s012.docx]

**Table S6 Nonsynonymous Variants of TNC in NSHL subjects**

| Chromosome position | Exon | cDNA position | Amino acid residue position | MAF ref.  (in dbSNP) | MAF detected  in our study | Subjects involved | SIFT  prediction | SIFT  score |
| --- | --- | --- | --- | --- | --- | --- | --- | --- |
| g.117853141C>T | 2 | **c.157G>A** | **p.V53I** | NA | A, 0.001241 | 1/403 | TOLERATED | 0.26 |
| g.117846580T>C | 4 | **c.2039A>G** | **p.Q680R** | C, 0.444 | C, 0.2254 | **77**/193 (include 10 C/C) | TOLERATED | 1 |
| g.117838716A>G | 8 | **c.2813T>C** | **p.V938A** | NA | C, 0.015 | **1**/33 | TOLERATED | 0.67 |
| g.117835899C>T | 10 | **c.3197 G>A** | **p.R1066H** | T, 0.118 | T, 0.064 | **61**/492 (include 2 T/T) | TOLERATED | 0.35 |
| g.117808785T>A | 17 | **c.5029A>T** | **p.I1677L** | A, 0.478 | T, 0.425 | **294**/436 (include 77 T/T) | TOLERATED | 1 |
| g.117792583C>G | 24 | **c.6022G>C** | **p.E2008Q** | C, 0.328 | C, 0.5597 | **432**/528 (include 159 C/C) | TOLERATED | 1 |
