# Supplementary material for: Exome Sequencing and Linkage Analysis Identified Tenascin-C (TNC) as a Novel Causative Gene in Nonsyndromic Hearing Loss
Source: PLoS One. 2013 Jul 30;8(7):e69549. doi: 10.1371/journal.pone.0069549 (PMC3728356; doi:10.1371/journal.pone.0069549)
Supplement: Table S7 — Nine Novel Nonsynonymous Variants of TNC detected in NSHL subjects. (DOCX) [file pone.0069549.s013.docx]

**Table S7** **Nine Novel Nonsynonymous Variants of TNC detected in NSHL subjects**

| Chromosome position | Exon | cDNA position | Amino acid residue position | MAF in our study | Subjects involved | SIFT score | SIFT prediction |
| --- | --- | --- | --- | --- | --- | --- | --- |
| g.117853264T>C | 2 | c.34T>C | p.F12L | T=2/818=0.002445 | 2/409 | 1 | TOLERATED |
| **g.117844143C>T** | **6** | **c.2312C>T** | **p.T771I** | **T=1/1148=0.0008711** | **1/574** | **0.05** | **DAMAGING** |
| g.117844115G>T | 6 | c.2340G>T | p.E780D | T=1/1148=0.0008711 | 1/574 | 0.14 | TOLERATED |
| **g.117840405G>A** | **7** | **c.2491G>A** | **p.D831N** | **A=3/1158=0.002591** | **3/579** | **0.02** | **DAMAGING** |
| g.117827064G>A | 11 | c.3349G>A | p.A1117T | A=1/842=0.001188 | 1/421 | 0.45 | TOLERATED |
| g.117826182G>A | 12 | c.3653G>A | p.R1218K | A=1/460=0.002174 | 1/230 | 0.48 | TOLERATED |
| **g.117822089C>G** | **14** | **c.3953C>G** | **p.A1318G** | **G=1/988=0.001012** | **1/494** | **0.02** | **DAMAGING** |
| g.117798506C>T | 21 | c.5527C>T | p.R1843C | T=1/1110=0.0009009 | 1/550 | 0.15 | TOLERATED |
| **g.117793879T>C** | **23** | **c.4781T>C** | **p.L1594P** | **C=1/1070=0.0009346** | **1/535** | **0.02** | **DAMAGING** |
| g.117792579 C>T | 24 | c.6026C>T | p.A2009V | T=3/968=0.003099 | 3/484 | 0.27 | TOLERATED |
