# Supplementary material for: Exome Sequencing and Linkage Analysis Identified Tenascin-C (TNC) as a Novel Causative Gene in Nonsyndromic Hearing Loss
Source: PLoS One. 2013 Jul 30;8(7):e69549. doi: 10.1371/journal.pone.0069549 (PMC3728356; doi:10.1371/journal.pone.0069549)
Supplement: Table S8 — Details of patients carried the deleterious novel variants. (DOCX) [file pone.0069549.s014.docx]

**Table S8 Details of patients carried the deleterious novel variants.**

| **ID** | **Age of onset (years)** | **Age of test (years)** | **Diagnosis** | **Variants** |
| --- | --- | --- | --- | --- |
| 400285 | 16 | 32 | auditory neuropathy | c.2312C>T;p.T771I |
| 501333 | 20 | 31 | sensoryneural hearing loss | c.2491G>A; p.D831N |
| 400347 | 18 | 23 | auditory neuropathy | c.2491G>A; p.D831N |
| 501217 | 13 | 15 | auditory neuropathy | c.2491G>A; p.D831N |
| 400286 | 1 | 11 | sensoryneural hearing loss | c.3953C>G;p.A1318G |
| 400734 | 12 | 47 | sensoryneural hearing loss | c.4781T>C;p.L1594P |
